# Setting the PAS, the role of circadian PAS domain proteins during environmental adaptation in plants

**DOI:** 10.3389/fpls.2015.00513

**Published:** 2015-07-09

**Authors:** Julia H. M. Vogt, Jos H. M. Schippers

**Affiliations:** ^1^Institute of Biochemistry and Biology, University of Potsdam, Potsdam, Germany; ^2^Institute for Biology I, RWTH Aachen University, Aachen, Germany

**Keywords:** PAS domain, circadian clock, signal transduction, environmental stress response, growth adaptation

## Abstract

The per-ARNT-sim (PAS) domain represents an ancient protein module that can be found across all kingdoms of life. The domain functions as a sensing unit for a diverse array of signals, including molecular oxygen, small metabolites, and light. In plants, several PAS domain-containing proteins form an integral part of the circadian clock and regulate responses to environmental change. Moreover, these proteins function in pathways that control development and plant stress adaptation responses. Here, we discuss the role of PAS domain-containing proteins in anticipation, and adaptation to environmental changes in plants.

## Introduction

The evolution of the photosynthetic apparatus resulted in two major developments on earth. During photosynthesis, water is split into oxygen (O_2_), protons and electrons, which caused the oxygenation of the earth’s atmosphere for about 2.2 billion years ago (Knoll, 2003; Schippers et al., 2012). The first event, the release of O_2_, placed organisms under strong selection pressure to mitigate the reactive nature of highly toxic O_2_ derivatives, i.e., superoxide, hydroxyl radical and hydrogen peroxide commonly called reactive oxygen species (ROS; Raymond and Segrè, 2006; Schippers et al., 2012). The release of oxygen stimulated the evolution of aerobic metabolism using a superior electron acceptor, resulting in an increased energy availability, which might have accelerated the development of multicellular organisms (Thannickal, 2009; Schirrmeister et al., 2013). The development of the photosynthetic apparatus itself gave rise to autotrophic organisms whose energy metabolism is light dependent. Considering the rotational behavior of the Earth, energy production in plants is limited to a specific time-window during a single day. Both, light dependent metabolism as well as redox oscillations, have stimulated the emergence of a control system that directs cellular processes in a temporal manner, the circadian clock (Lai et al., 2012; Haydon et al., 2013). Interestingly, the acquisition of aerobic metabolism, photosynthesis and the evolution of circadian systems appear to have co-occurred (Loudon, 2012).

At the molecular level, the circadian clock consists of several transcriptional feedback loops (Chow and Kay, 2013). The central loop is formed by the morning-expressed *CIRCADIAN CLOCK-ASSOCIATED1* and *LATE ELONGATED HYPOCOTYL* and the evening-expressed clock gene *TIMING OF CAB EXPRESSION1* (*TOC1*), which act in a reciprocal manner (Gendron et al., 2012). In principle, the transcriptional loops allow for activating or deactivating specific genetic programs at different times of the day. However, to ensure anticipation of environmental conditions and accurate timing of cellular processes, circadian clocks are set to the correct time of day through the perception of environmental signals (Harmer, 2009; Sanchez et al., 2011; Lai et al., 2012; Haydon et al., 2013). Light is the best-studied zeitgeber (a signal that resets the clock), that entrains or synchronizes the clock (Alabadí et al., 2001). In addition, other external cues such as temperature (Michael et al., 2003), but also internal cues like energy status and redox homeostasis (Lai et al., 2012; Haydon et al., 2013; Zhou et al., 2015), all feed back into the clock to optimize plant growth and survival (Dodd et al., 2005). Especially for the adaptation against adverse environmental conditions a functional clock is a prerequisite (Lai et al., 2012; Kolmos et al., 2014; Gehan et al., 2015). Moreover, genes associated with abiotic stresses show rhythmic expression behavior, and their transcriptional response often depends on the time of the day at which the plant encounters the stress (Gehan et al., 2015; Matsuzaki et al., 2015).

Plants, as sessile organisms heavily rely on their ability to sense and adapt to changes in their environment. One particular protein domain, the per-ARNT-sim (PAS) domain, is a sensory module that can be found in all kingdoms of life (Nambu et al., 1991). The PAS domain can function as a chemoreceptor, a redox sensor, a photoreceptor, or a voltage sensor (Möglich et al., 2009), indicating its versatile role in signal transduction. In animals, the PAS-domain containing ARNT transcription factor regulates the response to environmental stimuli by heterodimerization with different protein interaction partners to initiate a specific transcriptional response (McIntosh et al., 2010). Moreover, both in animals and plants, several of the circadian timekeeping proteins contain a PAS domain, forming a direct link between sensing the environment and the circadian clock. Here, we focus on the role of PAS domain proteins in environmental adaptation, under the control of the circadian clock.

## Structure of PAS Domains

**FIGURE 1:**
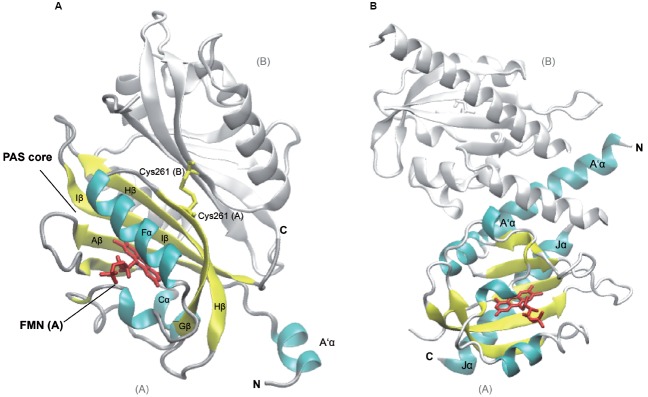
**Structures of *Arabidopsis thaliana* LOV1 and LOV2 domains of phototropin 1 dark-adapted state.** LOV1 domain (2Z6C; Nakasako et al., 2008), **(A)** and LOV2 of phototropin 1 in dark-adapted state (4HHD; Halavaty and Moffat, 2013), **(B)**. Subunits B of LOV1 and LOV2 are shown in white, subunit A of the respective LOV domains is shown in colors according to secondary structure. Several α-helices (cyan) and β-strands (yellow) of the PAS core are labeled according to their nomenclature within the PAS domain as well as flanking helices, which are involved in dimerization and signaling. The Flavin mononucleotide chromophores (red) and the disulfide-bond-creating Cys at position 261 (Cys261, yellow) in the monomers of LOV2 domains, respectively, are depicted as sticks. C- and N-termini of subunits A and B are labeled (C, N). The graphic representation of the structure was generated using VMD (Humphrey et al., 1996).

**FIGURE 2:**
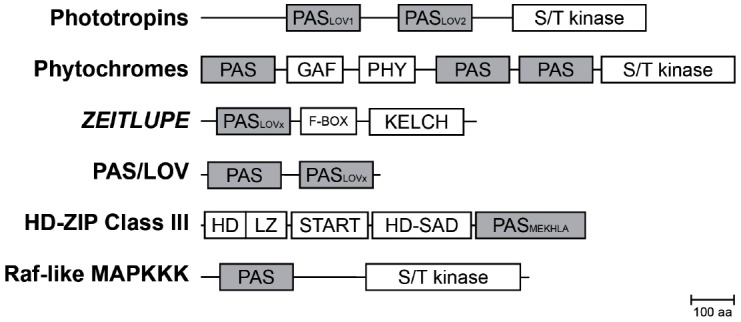
**Composition of PAS containing multidomain proteins in plants.** Schematic representation of PAS domain-containing proteins in *Arabidopsis* and their multidomain architecture (Mitchell et al., 2015). The PAS module is located in variable positions within the protein and associated with a variety of effector domains. Abbreviations: PAS, per-arnt-sim domain; PAS_LOV1/2_, LOV (light oxygen, or voltage) subclass of PAS domain; PAS_MEKHLA_, PAS-like MEKHLA domain; S/T kinase, Serine/Threonine kinase; F-BOX, domain interacting with SCF complex; KELCH, KELCH repeat; GAF, cGMP-specific phosphodiesterases, adenylyl cyclases and FhlA; PHY, GAF-like domain/Chromobillin binding; START, StAR-related lipid-transfer; HD-SAD, HD-START-associated domain; HD, homeodomain; LZ, Leucine zipper; aa, amino acids.

The PAS domain is a sensory and protein–protein-interaction module, which can be found throughout all kingdoms of life. It was originally identified by sequence homology of three eukaryotic proteins: the circadian protein Period (*per*) and developmental regulator Sim (*single-minded*) of *Drosophila* and the vertebrate *aryl hydrocarbon receptor nuclear transporter* (ARNT), which comprise two PAS motifs each (Nambu et al., 1991). Conserved residues C-terminal to the PAS motif were at first assigned as PAC motif (Ponting and Aravind, 1997). However, the first three-dimensional structure of the PAS domain revealed that both, the PAS and PAC motif, form a globular fold made up of about 100 residues, thus redefining the PAS domain (Hefti et al., 2004). Although PAS domains share only a low sequence homology on the amino acid level (∼20%), the three-dimensional structure is highly conserved (Mei and Dvornyk, 2014). The PAS fold consists of an antiparallel five-stranded β-sheet in topological order 2-1-5-4-3 and several flanking α-helices (Figure 1), which are either packed on the core or extend from it (Möglich et al., 2009). In plants, PAS domains are combined in multidomain proteins with functionally diverse effector/regulatory domains such as Serine/Threonine kinases, F-Boxes or, HD-ZIP domains (Figure 2 and Table 1), thus mediating a plethora of cellular responses. Interestingly, the phytochrome and F-BOX containing PAS domain proteins were shown to interact (Jarillo et al., 2001a; Kim et al., 2007) and function either as an input to the clock and/or an integral component of the circadian oscillator.

**Table 1:** **PAS-domain containing *Arabidopsis* proteins**.

**AGI**	**Protein**	**Function**	**References**
**Blue-light receptors**			
AT5G57360	ZTL	Regulation of circadian clock and photoperiodic flowering time	Somers et al. (2000)
AT3G18915	LKP2	Regulation of circadian clock and photoperiodic flowering time	Schultz et al. (2001)
AT1G68050	FKF1	Regulation of circadian clock and photoperiodic flowering time	Nelson et al. (2000)
AT3G45780	PHOT1	Phototropic response	Liscum and Briggs (1995)
AT5G58140	PHOT2	Phototropic response	Sakai et al. (2001)
AT2G02710	PLP	Involved in abiotic stress responses	Ogura et al. (2008a)
**Phytochromes**			
AT1G09570	PHYA	Seed germination, plant greening, cell expansion, and flowering time	Reed et al. (1994)
AT2G18790	PHYB	Seed germination, plant greening, cell expansion, and flowering time	Reed et al. (1994)
AT5G35840	PHYC	Seed germination, plant greening, cell expansion, and flowering time	Qin et al. (1997)
AT4G16250	PHYD	Seed germination, plant greening, cell expansion, and flowering time	Franklin et al. (2003)
AT4G18130	PHYE	Seed germination, plant greening, cell expansion, and flowering time	Delvin et al. (1998)
**HD-ZIP III transcription factors**			
AT3G34710	PHB	Meristem development and functioning; organ polarity and vascular development.	McConnell et al. (2001)
AT1G30490	PHV	Meristem development and functioning; organ polarity and vascular development.	McConnell et al. (2001)
AT1G52150	ICU4/CNA	Vascular development, meristem development; organogenesis	Ochando et al. (2008)
AT5G60690	REV	Meristem development and functioning; organ polarity and vascular development.	Talbert et al. (1995)
AT4G32880	ATHB8	Meristem development and functioning; organ polarity and vascular development.	Baima et al. (2001)
**MAPKKK**			
AT5G49470	RAF10	Positive regulators of ABA and seed dormancy	Lee et al. (2015)
AT1G67890	RAF11	Positive regulators of ABA and seed dormancy	Lee et al. (2015)
AT4G23050	MAP3K4	Involved in plant growth and abiotic stress responses	Sasayama et al. (2011)
AT3G06620	putative MAPKKK	Unknown	MAPK Group (2002)
AT3G06630	putative MAPKKK	Unknown	MAPK Group (2002)
AT3G06640	putative MAPKKK	Unknown	MAPK Group (2002)

### Structure of FMN-binding PAS-domain (LOV) in Plant Phototropins

The *Arabidopsis thaliana* genome contains two membrane-associated phototropin genes, *Phot1* and *Phot2* that encode serine/threonine kinases and serve as receptor for blue light (Kinoshita et al., 2001). They belong to a subclass of the PAS domain superfamily, termed LOV (light, oxygen, or voltage) domain. Defining feature of the LOV domain is the cofactor, a Flavin nucleotide (Huala et al., 1997). The photosensors harbor two N-terminal FMN-binding PAS domains, termed N- to C-terminal LOV1 and LOV2, which are coupled to the C-terminal kinase domain. The typical PAS fold consists of the five-stranded antiparallel β-sheet and four α-helices (β_2_α_4_β_3_: Aβ, Bβ, Cα, Dα, Eα, Fα, Gβ, Hβ, and Iβ; Figure 1). The core is flanked by amphiphatic α-helices (A’α, Jα) and mediates chromophore binding and signal transduction. Upon photoexcitation, the C4a of the oxidized FMN isoalloxazine ring forms a dark-reversible cysteyl-adduct with Cys39 within the LOV2 core domain (Salomon et al., 2000; Swartz et al., 2001) resulting in alteration of the hydrogen bonding in the binding pocket between the chromophore and the β-sheet, as well as the adjacent Jα helix (Crosson and Moffat, 2001; Halavaty and Moffat, 2013). Jα was shown to be required for kinase activity, and light-activated alterations of the interacting surface of the core domain leads to an intermediate partial unfolding of the α-helical structure. Together with the β-sheet it might constitute the interface involved in signal transmission by alleviating the repressive effect on the protein kinase domain of phototropins (Crosson and Moffat, 2002; Crosson et al., 2002; Corchnoy et al., 2003; Harper et al., 2003; Matsuoka and Tokutomi, 2005; Chen et al., 2007; Pfeifer et al., 2010). A’α and Jα helices both contribute to coiled coil dimerization of the phototropin (Figure 1; Halavaty and Moffat, 2013).

The two PAS cores share a high degree of similarity among each other and among the phototropins (Halavaty and Moffat, 2007; Nakasako et al., 2008) and exhibit identical photochemical cycles (Salomon et al., 2000; Kasahara et al., 2002). However, there are functional differences between the two domains: while LOV2 is required and sufficient for light sensing and the autophosphorylation resulting in phototropic hypocotyl growth (Christie et al., 2002), the LOV1 domain, mainly contributes to dimerization and regulating the sensitivity to blue-light activation (Matsuoka and Tokutomi, 2005; Nakasako et al., 2008; Tokutomi et al., 2008). The dimerization sites of LOV1 in the two phototropins are similar to the extent that they both form a face-to-face association of the β-scaffolds. They are stabilized by hydrophobic interactions of an N-terminal α-helix (relative to the PAS core) with the β-scaffolds of both subunits (Nakasako et al., 2008). While the LOV1 dimer of Phototropin 1 forms a stable disulfide bond [Cys261(A)—Cys261(B), Figure 1A], LOV1 of Phototropin 2 dimer is predominantly stabilized by hydrogen bonds of a threonine and methionine (Nakasako et al., 2008), underlining the structural rather than sequence conservation and giving a possible explanation for the different physiological roles of the two phototropins in plants.

### Sensing-signaling Mechanisms

It was recently postulated that the general role for PAS domains is to modulate protein–protein interactions to form hetero- or homo-oligomers with other proteins (Möglich et al., 2009), or alter protein structures by rearranging interactions between domains in a single protein. As the PAS domain can act as a sensor, the occurrence of protein–protein interactions becomes signal dependent. This property provides specificity, allowing for complex spatial and temporal regulation of cellular signaling networks (Möglich et al., 2009). Here, the role of PAS domains in the modulation of protein activity and signaling is illustrated by several examples.

In contrast to other species, plant phytochromes contain three PAS domains (Hughes, 2013). It has been shown for PhyB, the most prominent photo-stable phytochrome, that the nuclear translocation depends on the tandem PAS domain (Chen et al., 2005). In its inactive form the tandem-repeat PAS domain interacts with the N-terminal sensory domain containing the other PAS motif (Figure 2). Upon red light, the protein is activated through a conformational change, exposing the nuclear-localization signal present in the tandem PAS motif, resulting in translocation to the nucleus. In addition, the tandem PAS domain is crucial for the red-light induced interaction between PhyB and PHY INTERACTING FACTORs (PIFs; Levskaya et al., 2009). Moreover, application of far-red light results in a reversal of the interaction between PhyB and PIFs (Ni et al., 1999). This photoreversibility of the protein interaction depends on the presence of the tandem PAS domain (Levskaya et al., 2009). Still, sensing of red light by phytochromes is established through the billin chromophore bound by the GAF domain within the N-terminal photosensory module (Galvão and Fankhauser, 2015). Although the function of the PAS domain in the photosensory module is unknown, it was shown to interact with and thereby stabilizing the chromophore binding GAF domain, which has also been implicated in PIF binding (Burgie et al., 2014). Thus, the PAS domains in phytochromes do not function as light sensors, and it is currently unclear, whether they have sensory roles themselves or mainly support conformational changes (Burgie and Vierstra, 2014).

The phototropins and ZEITLUPE family of LOV domain-containing proteins act as blue-light receptors. The ZTL family contains an N-terminal LOV domain followed by an F-box and six Kelch repeats (Figure 2), suggesting a role in light-regulated protein degradation (Demarsy and Fankhauser, 2009). F-box proteins are components of SCF-type (Skp1, Culin, and F-box) ubiquitin E3 ligases and ZTL was shown to interact with *Arabidopsis* Skp-like (ASK) proteins (Kevei et al., 2006). A mutation in the LOV domain of ZTL, as found in the *ztl-21* mutant, was shown to interfere with the interaction with ASK1 (Kevei et al., 2006), indicating that light signals are required for ZTL to target proteins for degradation. In the light, photoexcitation of ZTL promotes interaction with GIGANTEA (GI), stabilizing ZTL during the day (Kim et al., 2007). Still, ZTL is not completely stabilized as photochemical characterization of ZTL reveals a half-life of about 4 h (Pudasaini and Zoltowski, 2013). It was postulated that this fast photocycle allows for estimating the timing of the day-night transition. Thus, the LOV domain of ZTL determines protein stability and directs protein–protein interactions.

So far, only a role for the PAS sensory domain in light receptors has been described, in other plant proteins the function of the PAS domain is mostly unknown and the signals they perceive are elusive. For example, the HD-ZIP III family of transcription factors contains a C-terminal PAS-like domain. A point mutation in this domain for CORONA/INCURVATA4 results in a bushy plant phenotype, suggesting a regulatory role for this domain (Duclercq et al., 2011). Furthermore, the PAS-like domain in HD-ZIP III member REVOLUTA (REV), was shown to modulate the DNA binding activity of the protein (Xie et al., 2014), however, the signal that modulates the regulatory function of the PAS-like domain in this case is unknown. Therefore, additional efforts are required to better understand the role of PAS domains in plants.

## Plant PAS Domain Proteins

Plants as phototrophic sessile organisms are required to adapt especially to changes in light, but also other environmental cues. Hence, it is not surprising that many of the PAS sensory modules are coupled to light perception and signaling (Table 1). Among the five classes of photoreceptor families characterized in *Arabidopsis*, the phytochrome, cryptochrome, and ZEITLUPE family integrate the red/far-red and blue light information into the central oscillator (McClung, 2011).

### Phototropin 1 and 2, Tandem LOV Domain Blue-light Receptors

The plant specific blue-light (and UVA) receptor protein kinase Phot1 was initially identified by a forward genetic screen for plants showing defects in the phototrophic response (Liscum and Briggs, 1995; Huala et al., 1997). Moreover, phototropins 1 and 2 are involved in blue-light induced stomata opening, and chloroplast migration (Jarillo et al., 2001b; Kinoshita et al., 2001; Sakai et al., 2001). This indicates that both receptors play an important role in sensing the light availability, for metabolic and physiological adaptation.

The structure of the two phototropins have been extensively studied (Christie et al., 2002; Nakasako et al., 2008; Halavaty and Moffat, 2013) and helped understanding the underlying mechanism of the structure-function relationship of PAS (LOV) domains. In the dark, each LOV domain non-covalently binds FMN, while blue light causes covalent binding of the chromophore, resulting in the release of its inhibitory effect on the C-terminal kinase domain — thereby facilitating autophosphorylation and subsequent activation of the phototropin signal transduction pathway (Tokutomi et al., 2008). Of note, this reaction is reversible allowing for a rapid switch of phototropin activation in a light-dependent manner.

Recently it was found that PIF4 and PIF5 act downstream of Phot1 to negatively modulate phototropism in *Arabidopsis* (Sun et al., 2013). Although no direct link between the circadian clock and Phot1/2 has been established, the dependence of PIF4 and PIF5 on Phot1 might present such an interaction. *PIF4* and *PIF5* expression are under the control of the evening complex, containing the proteins EARLY FLOWERING 3 (ELF3), ELF4, and LUX ARRHYTHMO (LUX) during hypocotyl elongation (Nusinow et al., 2011). Still, this interaction has so far not been described.

### Phytochromes, Tandem PAS Domain Red-light Receptors

Phytochromes act as homodimeric red and far-red photoreceptors that are encoded by a small family of genes encompassing three clades, PHYA, PHYB, and PHYC (Casal, 2013). As stated above, they contain a single PAS motif within their phytochrome sensory domain, which is followed by a tandem PAS domain and histidine-kinase-like transmitter module (Hughes, 2013). In plants, phytochromes regulate a multitude of processes, including, seed germination, plant greening, cell expansion, and flowering time to regulate plant growth and development in response to environmental light cues (Galvão and Fankhauser, 2015).

Of note, PhyB mutants display a long circadian period, predominantly under continuous red light, while PhyA mutants display long period under constant red and blue light (Somers et al., 1998). PhyB has been shown to directly interact with ELF3 (Liu et al., 2001), forming a link to the clock. In addition, PIFs were found to negatively regulate PhyB levels by promoting the proteasome dependent degradation of the activated PhyB form (Jang et al., 2010; Ni et al., 2014). Interestingly, it was found that nuclear-localized PhyB is required for stabilizing the ELF3 protein (Nieto et al., 2015), which represses the PIF4 function by sequestering the protein. Moreover, PhyB is known to interact with another PAS domain containing clock protein, ZTL/ADO1 (Jarillo et al., 2001a), as described below. In contrast, no direct interaction between the other four phytochrome proteins with clock proteins have been reported so far.

### Circadian Clock Proteins ZTL, LKP2, and FKF1

The three paralogs in *Arabidopsis*, ZEITLUPE (ZTL), FLAVIN-KELCH-FBOX-1 (FKF1), and LOV-Kelch-Protein-2 (LKP2), share a high degree of amino acid identity of about 70–80% (Somers et al., 2000). They comprise a single N-terminal LOV-type PAS domain similar to that of phototropins binding oxidized FMN (Imaizumi et al., 2003), followed by an F-Box and a C-terminal Kelch-repeat domain, which mediate formation of SCF E3 ligase complexes and substrate binding for polyubiquitination, respectively (Somers et al., 2000; Andrade et al., 2001; Yasuhara et al., 2004; Figure 2). Blue light is perceived in a fluence-dependent manner via the PAS (LOV) domain and fed to the light-input of the circadian clock and regulation photoperiodic flowering control via protein–protein-interactions (Nelson et al., 2000; Imaizumi et al., 2003; Somers et al., 2004; Takase et al., 2011). The protein–protein-interaction either targets the partner to proteasomal degradation, such as the core clock components TOC1 and PRR5 by ZTL in the dark, or stabilizes the proteins, as shown for interactions of FKF1 with CONSTANS (CO) and CYCLING DOF FACTORS (CDFs), or all three proteins with GIGANTEA (GI) in blue light (Imaizumi et al., 2005; Kiba et al., 2007; Kim et al., 2007; Baudry et al., 2010; Song et al., 2014). Despite their structural similarity, ZTL, FKF1, and LKP2 exert distinct physiological roles. While ZTL is a major player in the evening loop of the circadian clock, the latter are mainly involved in the morning loop. This is achieved by a combination of different regulatory mechanisms, such as differential expression, protein stability and intracellular localization, as well as the photokinetic properties and fluence sensitivities of their light-sensing PAS domains (Somers et al., 2000, 2004; Kim et al., 2007; Baudry et al., 2010; Pudasaini and Zoltowski, 2013; Song et al., 2014).

ZTL was shown to interact with PhyB *in vitro* (Jarillo et al., 2001a), and several mutations in either of the domains of ZTL protein, including the PAS domain, did not abolish the interaction with the C-terminal part of PhyB, indicating that *ztl* phenotypes are not due to disrupted interaction between these two proteins (Kevei et al., 2006).

### PAS/LOV Protein—Another Potential Plant Blue-light Receptor

*Arabidopsis* encodes yet another potential photoreceptor, termed PAS/LOV protein (PLP isoforms A to C). PLP harbors a PAS domain at either end of the protein, the C-terminal domain showing homology to FMN-binding LOV domains. Having no predicted effector domain, however, it is likely that they act *in trans* on other proteins (Crosson et al., 2002).

Although PLPs have not been studied in great detail and the exact function of both PAS domains are not resolved to date, they seem to be involved in the plant reaction to salt and dehydration stress, and protein interaction is modulated in a blue-light dependent manner in yeast (Ogura et al., 2008a,b). In addition, the *PLP* gene shows a clear diurnal expression pattern, with a maximum expression in the afternoon (Mockler et al., 2007), suggesting that it might act as a clock output.

### PAS-like Domain in HD-ZIP Transcription Factors

REVOLUTA (REV), PHABULOSA (PHB), PHAVOLUTA (PHV), INCURATA4/CORONA (ICU4/CAN), and ATHB8 (ARABIDOPSIS THALIANA HOMEOBOX PROTEIN 8) constitute the plant-specific Class III of homeodomain leucine zipper (HD-ZIP III) transcription factors. They are well studied major regulators of developmental processes and involved in establishing adaxial-abaxial polarity in lateral organs, meristem regulation and formation, vascular development, as well as embryonic patterning (Talbert et al., 1995; Zhong and Ye, 1999; McConnell et al., 2001; Otsuga et al., 2001; Prigge et al., 2005; Carlsbecker et al., 2010; Smith and Long, 2010). As a recurring pattern in plant PAS domain proteins of a family, they function not only redundantly, but display distinct, yet antagonistic function (Prigge et al., 2005).

The multidomain proteins consist of the N-terminally located eponymous HD-ZIP domain followed by two regulatory domains, the lipid ligand binding START and the START associated domain (HD-SAD, Mukherjee et al., 2009; Schrick et al., 2014). START/HD-SAD domains serve as regulatory domains recognized by microRNAs miR165 and miR166 (Emery et al., 2003). The C-terminus is constituted by a so-called MEKHLA domain, an approximately 150 amino acid long PAS-like domain (Mukherjee and Bürglin, 2006) that shares highest homology to bacterial sensor histidine kinases such as the oxygen sensor FixL (Hao et al., 2002; Mukherjee and Bürglin, 2006). It is not known, however, whether the transcription factors bind the same cofactor (i.e., heme) with its PAS-like domain because of the high sequence variability (Mukherjee and Bürglin, 2006). The PAS-like domain negatively regulates REV activity by inhibiting protein homodimerization (Magnani and Barton, 2011). Upon stimulation this inhibition is lifted and the REV protein is activated. Of note, HD-ZIP III proteins are known to be redox-sensitive (Comelli and Gonzalez, 2007; Xie et al., 2014), suggesting that the PAS-like domains in these proteins might also sense oxygen or ROS. A genome-wide binding analysis for REV revealed that *PRR5* is a target gene, representing a link with the morning-loop of the circadian clock (Brandt et al., 2012).

### MAPKKKs Harboring PAS Domains

Mitogen-activated protein kinase (MAPK) cascades are a common way of signal transduction and they are associated with integrating environmental cues into adequate responses (Romeis, 2001; Sinha et al., 2011). *Arabidopsis* encodes about 80 MAPKKK (MAP3K) genes. The subgroup B2 of Raf-like kinases consists of six members and comprises an N-terminal PAS domain (MAPK Group, 2002; Colcombet and Hirt, 2008). To date, knowledge of the physiological framework in which this subgroup of MAPKKKs exerts its role, the signaling mechanism and molecular function of the PAS domain is scarce.

MAP3Kδ4 was reported to be an auxin- and abscisic acid (ABA)-responsive kinase affecting plant growth, mediating tolerance to salt stress, and it is likely to be involved in other abiotic stress responses as well (Sasayama et al., 2011; Shitamichi et al., 2013). Recently, two functional redundant putative MAPKKK of the same group, Raf10 and Raf11, were identified likewise as positive regulators of ABA response and seed dormancy (Lee et al., 2015). As for MAP3Kδ4, the exact molecular function of the PAS domain within the MAPK signaling is not yet understood. Moreover, in animals it has been shown that a MAPK cascade can reset the circadian clock (Akashi and Nishida, 2000), whether such a scenario also exists in plant remains to be discovered.

## Environmental Sensing and Plant Adaptation Through PAS Domain-containing Proteins

Light is the major clock input signal, which is used by plants to set the pace and phase of the oscillator, adjusts growth and development to changing environmental conditions. Most of the here described PAS domain-containing proteins sense light, and have been shown previously to be involved in entraining the circadian clock.

Although the PAS domain-containing phytochromes represent an important class of photoreceptors, they are not required for regulating circadian responses, *per se* (Yanovsky et al., 2000; Strasser et al., 2010). Still, under free-running conditions, in a light-intensity and quality dependent manner, phytochrome mutants were shown to exhibit a lengthened or reduced period and arrhythmic leaf movements (Somers et al., 1998; Strasser et al., 2010). Although light input is essential for clock periodicity, it has not been possible so far to relate this strongly to the presence of phytochromes, even though PhyB interacts with and stabilizes ELF3 in the light (Nieto et al., 2015). Potentially, the loss of phytochrome-dependent light signaling can be compensated for by other photoreceptors to sustain light entrainment of the clock. Indeed, it was previously shown that cryptochromes mediate phytochrome signaling to the clock in both red and blue light (Devlin and Kay, 2000). In addition, it has been established that the blue light cryptochromes differentially control circadian period and sustain rhythmicity across a physiological temperature range (Gould et al., 2013). Still, PhyB and ELF3 were shown to be negative regulators of dark-induced senescence (Sakuraba et al., 2014). Prolonged light-deprivation causes the inactivation of PhyB and the subsequent accumulation of PIF4 and PIF5 and the onset of leaf senescence. Under normal day-night rhythms, ELF3 represses *PIF4* and *PIF5* expression during the night. However, prolonged darkness overrides this control. Interestingly, short pulses of red light during 10 days of darkness prevent the onset of senescence in a PhyB dependent manner (Sakuraba et al., 2014).

The LOV domain-containing ZTL was shown to interact with PhyB as well as with CRY1, linking both phytochrome and cryptochrome with the circadian clock (Jarillo et al., 2001a). The *ztl* mutant is described as a clock mutant with altered period in a light-dependent manner (Somers et al., 2000). Therefore, ZTL might regulate the integration of light signals into the clock. In *Arabidopsis*, the blue-light receptor CRY2 interacts with and activates the transcription factor CRYPTOCHROME-INTERACTING basic helix–loop–helix 1 (CIB1). CIB1 regulates the floral integrator gene *FLOWERING LOCUS T* (*FT*), which in turn controls floral initiation (Liu et al., 2008). In blue light, ZTL/LKP2 protect CIB1 from proteasomal degradation (Liu et al., 2013) emphasizing the convergence of more than one, evolutionary distinct PAS domain-containing light receptors involved in photoperiodic regulation. Next to a role of CIB1 in regulating flowering time, it was found to have a negative impact on plant immunity (Malinovsky et al., 2014). In line with this, ZTL was found to affect the plant defense response (Wang et al., 2011).

In addition, the ZTL family target PRR5 was shown to repress the expression of genes involved in abiotic stress (Nakamichi et al., 2009, 2012). Furthermore, a triple loss of function *prr5 prr7 prr9* mutant displays enhanced cold, drought and salinity tolerance, placing the clock PRR proteins in a central position mediating adaptation to environmental cues. Interestingly, overexpression of LKP2 improves plant drought tolerance by positively regulating dehydration-inducible genes such as DREB1A-C, RD29A, as well as DEHYDRIN (Miyazaki et al., 2015). Moreover, these genes overlap with those that show elevated expression in the *prr5 prr7 prr9* mutant (Nakamichi et al., 2012), in agreement with a role of LKP2 in promoting the degradation of PRR5 (Baudry et al., 2010), and thereby promoting plant adaptation to a changing environment.

The HD-ZIPIII transcription factor REV was shown to control oxidative stress tolerance in *Arabidopsis* (Xie et al., 2014), aside from its role in plant development. This indicates that the HD-ZIP III family might be an important player integrating environmental signals for growth regulation. However, still little is known regarding the emerging role of this family in adaptive growth. Initial findings suggest a rather complex network embedding the transcription factors requiring thorough future investigations.

## Conclusion

The PAS domain fold is a common protein module found throughout the kingdoms of life. Although the domain has been implicated in sensing a multitude of signals, in plants only evidence for a role in light signaling has been obtained so far. Many of the PAS domain-containing proteins are intimately connected to the circadian clock, suggesting that they might contribute to linking environmental conditions with clock entrainment.

Here, we presented the emerging links between PAS domain proteins, time keeping and the response of the plant to the environment. So far, a clear implication for light signaling and plant adaptation through PAS domain proteins has been established by predominantly altering protein–protein interaction properties of their proteins. Still, plenty of effort is needed to dissect the biochemical and mechanistic functions of most PAS domain proteins in plants. The role of most of these proteins in response to the environment is unexplored. In addition, the stimulus upon which they exert their function remains elusive. An appealing aim would be to characterize the family of MAPKKK proteins further, which seem to be linked with abiotic stress tolerances. It would be intriguing to reveal whether the PAS domain controls their signaling activity.

### Conflict of Interest Statement

The authors declare that the research was conducted in the absence of any commercial or financial relationships that could be construed as a potential conflict of interest.
